# Pilot Cadaveric Study of Anatomical Variations of the Median Nerve at the Wrist in the Lithuanian Population

**DOI:** 10.7759/cureus.39282

**Published:** 2023-05-21

**Authors:** Markus Neumann, Andrej Suchomlinov

**Affiliations:** 1 Department of Anatomy, Histology, and Anthropology, Vilnius University Faculty of Medicine, Vilnius, LTU

**Keywords:** anatomy at the wrist, thenar motor branch of median nerve, median nerve, carpal tunnel, anatomical variations

## Abstract

Introduction: Carpal tunnel syndrome (CTS) is known as one of the most common neurological disorders in the human body. Nowadays, the prevalence in the general population ranges between 1% and 5%. Due to its high prevalence and increasing incidence of carpal tunnel surgery, the anatomical variations of the median nerve at the wrist are important to know to avoid iatrogenic injury of the nerve.

Purpose: The objective of this study was to evaluate the anatomical variation of the median nerve at the level of the wrist in the Lithuanian population with a focus on its thenar motor branch based on the classifications of Lanz.

Material and methods: A cadaveric study was performed, and 30 wrists of 15 adult Lithuanian cadavers ranging from 70 to 89 years of age were dissected and examined. Eight female and seven male cadavers were included in the study. Any anatomical finding was documented, and the results were compared with the classification of Lanz as well as with the data found in the literature.

Results: All hands showed different patterns in comparison to the standard anatomical variation Lanz type 0. The most common result was dedicated to Lanz group 4A. Nineteen out of 30 hands (63%, p<0.01) had an accessory branch proximal to the carpal tunnel, while one of these hands showed a third thenar motor branch. Five hands (16%) were dedicated to Lanz group 2 with an accessory branch distal to the carpal tunnel. One hand (3%) showed a variation close to Lanz group 2, but in this case, the thenar motor branch had its origin under the flexor retinaculum instead of proximal to it. Two hands each (6%) were classified by Lanz groups 1B and 3A. Additionally, one variation showed a pattern of a combination of Lanz types 3A and 3B. The bifid median nerve had a connecting branch in between which started distal to the flexor retinaculum. Two anatomical variations (6%) were not described by the classification of Lanz.

## Introduction

Carpal tunnel syndrome (CTS) is known as one of the most common neurological disorders in the human body. It was already described in the 19th century [1]. Nowadays, the prevalence in the general population ranges between 1% and 5% [2,3]. The etiology of CTS is due to compression of the median nerve between the flexor retinaculum (FR) and other structures within the carpal tunnel (CT). This can be caused by synovitis, tumors, tissue infiltration, congenital syndromes and systemic diseases [3,4]. Due to its high prevalence, CTS is also the most expensive upper-extremity musculoskeletal disorder in the US. Annually, the estimated cost of medical care primarily for carpal tunnel release (CTR) surgery exceeds $2 billion, while the non-medical costs are essentially higher [2].

Similar findings are reported by Tadjerbashi et al. [4] published in Sweden, in 2019. The purpose of their study was to investigate the change in the incidence of referred CTS and CTR. Within eight years (2001-2009), the incidence per 100,000 person-years of diagnosed CTS increased in both sexes. The mean increase in women per year was 1.8% and in men 3.9%. The incidence of CTR surgery increased as well. The mean annual increase in women was 5.1% and 6.2% in men [5].

Since anatomical variations at the wrist are rather frequent, the knowledge of possible variations to avoid iatrogenic injury during surgery is mandatory [6-8]. Even though, the CT and its contents may be visualized by sonography during or before surgery, the incidence of iatrogenic injuries is still quite high. Iatrogenic nerve injuries account for about 20% of traumatic nerve lesions [7]. This injury used to be called the “million-dollar injury” because of the high costs of compensation for the disability to use the thenar muscles properly [9].

The Neurosurgical Department of the University of Ulm performed a study over a period of 23 years from 1990 to 2012. The study included 340 patients who were treated for iatrogenic nerve injuries. Antoniadis et al. [7] published that the most commonly affected nerve was the median nerve in 17% of the cases (54 out of 340), followed by the accessory nerve in 16%. Together, the nerves at the wrist, radial nerve (RN), ulnar nerve (UN) and median nerve (MN), represented almost 40% (131 out of 340) of the biggest group for iatrogenic nerve injury. Additionally, the number of iatrogenic nerve injuries increased over the years. The number of affected patients increased from 2000 to 2007 from 10 to 27 [7].

This pilot cadaveric study deals with the anatomical variations of the CT in the Lithuanian population with a special focus on the MN and its branches. All variations we found were compared and classified according to the schematic illustrations of Lanz. Furthermore, the results and the prevalence of our results were compared to other studies. Additionally, the classification of Poisel is important for general understanding since some studies include the classification of Poisel while comparing their results to other studies [10]. More detailed information regarding these classifications will be explained in this paper.

The anatomical borders of the CT are made up of the scaphoid bone radially, the lunate and capitate bones dorsally and the hook of the hamate bone on the ulnar site [10]. The FR builds the superficial border. In total, 10 structures are passing through the CT: four tendons of the flexor digitorum superficialis (FDS) and four tendons of the flexor digitorum profundus (FDP). Additionally, the tendon of the flexor pollicis longus (FPL) and the MN are passing through the CT [10]. Normally, the MN is located between the FR and the FDS, but its position within the CT may vary [11].

One of the first branches of the MN at the wrist is the thenar motor branch (TMB). Usually, the TMB arises from the radial site of the MN. In very rare cases (about 2%), the TMB originates from the ulnar site of the nerve [12]. The TMB is supplying most of the three thenar muscles on the radial aspect of the hand before terminating in its sensory branches.

These muscles include the abductor pollicis brevis, opponens pollicis and a partial innervation of the flexor pollicis brevis [13]. However, the prevalence of anatomical variations of the MN and especially of its branches at the wrist varies in the literature. Lanz reported in a study including 246 hands that in 46% of cases, the MN had an extraligamentous branch (group 0) [5]. A subligamentous branch was found in 31% (group 1A) of cases, and a transligamentous branch was least common in 23% (group 1B) [6]. Further anatomical variations of the MN were reported in 29 hands (12%). The most frequent variation found in 18 hands (7%) was group 2 with an additional accessory branch. A high division of the MN was found in seven hands (3%) which are dedicated to group 3. The least common variation found in four hands (1.6%) was group 4 with an accessory branch proximal to the FR [6]. Compared to Agarwal et al., the most common variation was a transligamentous branch in 22 out of 52 (42.3%) hands, while the extraligamentous branch was the second most common variation with about 36% [14].

In a meta-analysis published in 2015, 31 studies (n=3,918 hands) were included. The prevalence of extraligamentous, subligamentous and transligamentous was 75.2%, 13.5% and 11.3%, respectively. The variation of Lanz group 2 was found in 4.6% of cases and Lanz group 3 in 2.6%. Lanz group 4 had a prevalence of 2.3% [9]. Beris et al. reported anatomical variations of the MN in 10% of the cases in a study including 110 patients. Groups 1A and 2 were found in three cases (2.7%). Two cases each (1.8%) were dedicated to groups 1C and 3B [15]. Steinberg et al. dissected 46 hands in a study published in 1998. They reported a prevalence of the anatomical standard variation in 33 hands (71.7%). In 10 hands (21.7%), an accessory TMB was found which is classified by Lanz group 4A [16]. Three hands were dedicated to Lanz group 1B.

## Materials and methods

The study was performed at the Department of Anatomy, Histology and Anthropology of Vilnius University Faculty of Medicine, Lithuania. The literature review was carried out by mainly using PubMed, EMBASE, Science Direct, Cochrane Library and Scopus database. The keywords included median nerve, nervus medianus, thenar motor branch, anatomical variations, carpal tunnel, flexor retinaculum, transverse carpal ligament, extraligamentous, subligamentous, transligamentous, high division, recurrent branch, accessory branch and anatomy. The comprehensive search was performed in the English language. A filter was used to search for literature most recently published. Since there were a lot of studies (including cadaveric studies) published more than 20 years ago, they were considered as well. One example is the clinical trial and classification of Lanz which was published in 1977. Basically, only other cadaveric studies, intraoperative studies, reviews and meta-analysis were used to compare the results of this study. The tables were created by using Microsoft Excel (Microsoft Corporation, Redmond, Washington, USA) and SPSS (IBM Statistics, IBM Deutschland GmbH, Ehningen, Germany). The results were transferred to SPSS (IBM) and statistically analyzed. The data were checked for normal distribution. A descriptive statistical analysis of the proportion and a test for its statistical significance, a parametric p-value test, has been performed.

For this study, 15 cadavers were included and donated to the Department of Anatomy, Histology and Anthropology of Vilnius University of Medicine, Lithuania. In total, eight female and seven male cadavers were dissected over the period of one year. A dissection protocol was created. Every cadaver was given a code based on the consecutive number of dissection and the side (left or right arm) of dissection. Furthermore, the anatomical standard content of the carpal tunnel was included as well as commonly known variations. Additionally, the classifications of Lanz were included. The age as well as the sex was written down. All our donors have signed special notaries’ approved forms that let conduction of scientific research with their bodies. The forms may be provided upon request (however, they are in Lithuanian).

To perform a correct dissection of the cadavers, the book “Grant's Dissector” was used before the first dissection started [17] and approval from the ethical committee was given. Each of the individuals included in the study while still being alive has given permission to conduct scientific research with their bodies after death. There were no known cases of congenital anomalies, deformities or injuries at the wrist.

**Figure 1:**
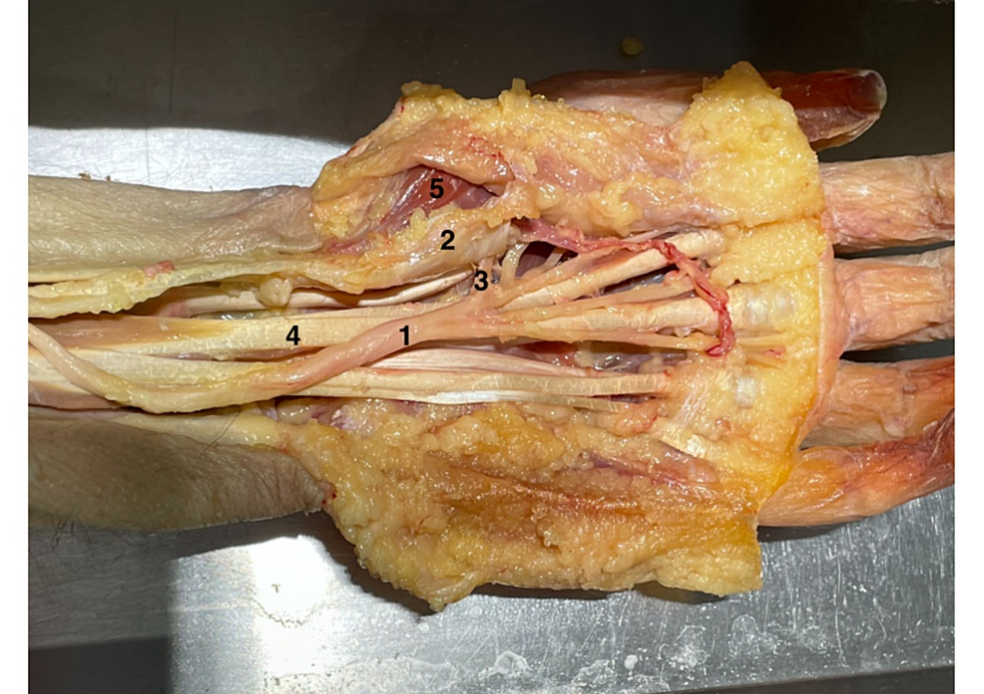
Dissection method Dissection method: 1) MN; 2) FR; 3) TMB; 4) tendons of FDS; and 5) thenar muscles. MN: median nerve, FR: flexor retinaculum, TMB: thenar motor branch, FDS: flexor digitorum superficialis.

A longitudinal skin incision was made from the distal forearm across the palm to proximal interphalangeal joints. Afterward, a transverse incision on the anterior surface of digits two to five was performed. The superficial palmar fascia as well as subcutaneous tissue and fat were carefully removed from the flexor retinaculum. A probe was inserted from proximal to distal deep to the flexor retinaculum. The ulnar approach to the carpal tunnel was performed by an anterior longitudinal cut with a scalpel along the probe. After identifying the median nerve (MN), its thenar motor branches (TMBs) and the content of the carpal tunnel photos were taken and any variation if present compared to the schematic illustrations of Lanz classification (Figure 1).

Lanz classification

**Figure 2:**
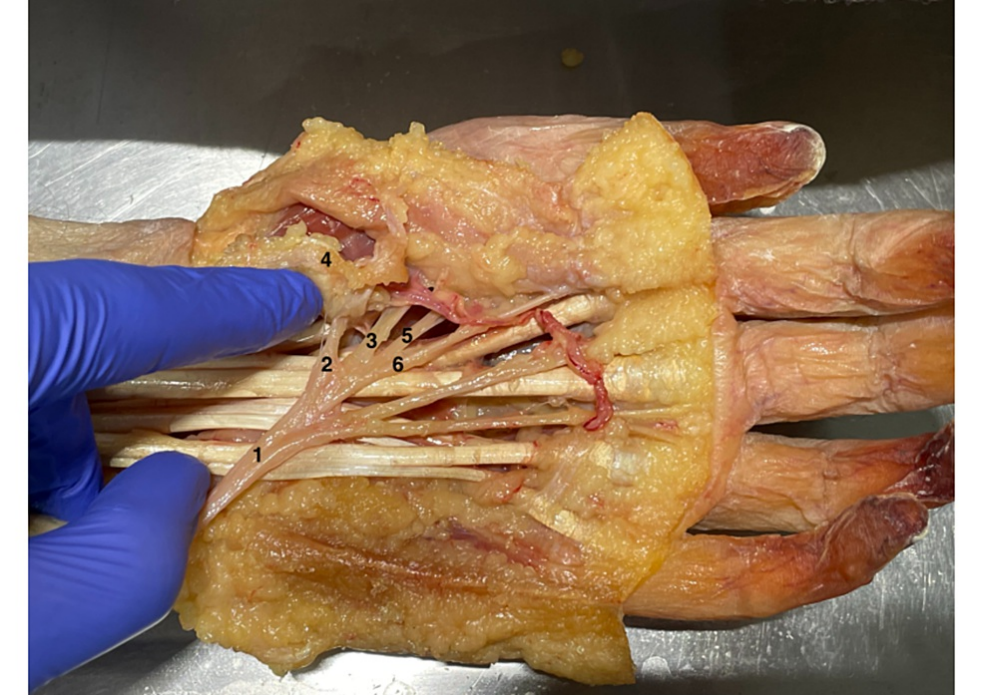
Lanz Group 4A Lanz group 4A: 1) MN; 2) transligamentous TMB; 3) accessory TMB; 4) flexor retinaculum; and 5), 6) MN branches to the index finger. MN: median nerve, TMB: thenar motor branch.

As mentioned before, to understand a detailed description of the classification of Lanz, the classification of Poisel is important for general understanding. Some studies include the classification of Poisel while comparing their results to other studies. Poisel distinguished basically three types of branching of the TMB in relation to the FR: the extraligamentous distal to the FR (type I), the subligamentous which arises within the CT and remains deep till the end of FR without piercing the FR (type II) and the transligamentous type which pierces the FR (type III) [10]. The classification of Lanz is a more extended and detailed version of this classification and consists basically of four different groups: group 1, group 2, group 3 and group 4 (Figure 2) which are then again subdivided according to their specific variation and branching.

Variations belonging to group 1 are describing the course of one single TMB: extraligamentous, transligamentous, subligamentous and ulnawards. Group 2 describes an additional accessory branch of the MN at the distal CT, while group 4 includes the MN and its accessory branches proximal to the CT. Anatomical variations with a high division of the MN with or without a connecting branch, without artery and without lumbrical muscles having their origin in the CT, are represented by Lanz group 3A. Lanz group 3B describes a high division of the MN and additionally includes a persistent median artery. Anatomical variations with a high division of the MN with a connecting branch and lumbrical muscles having their origin within the CT are dedicated to Lanz group 3C.

## Results

Eight female and seven male cadavers were examined. In total, 15 cadavers were dissected. In all cadavers, the FR was present and not cut before. The most common variation found was Lanz group 4A (Figure 2). In over 63% of cases (19 hands), an additional accessory TMB proximal to the FR was found. The distribution of this variation in the hands was quite equal on both sides. In 10 cases, the variation was found in the left arm, while nine were found in the right arms.

**Figure 3:**
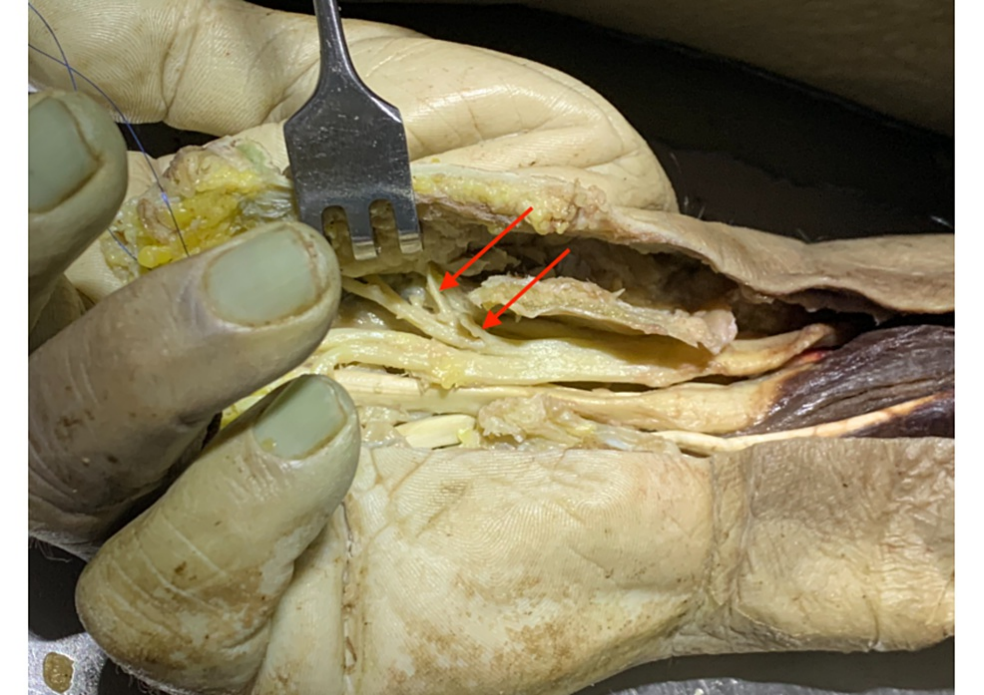
Lanz group 2 with two TMB, one with the origin under and one distal to the FR First TMB distal to the FR (right red arrow), and second TMB distal to the FR (left red arrow). TMB: thenar motor branch, FR: flexor retinaculum.

The second most common variation in 16.6% of cases was Lanz group 2 (Figure 3). In five hands, two TMBs distal to the FR were found. In two cases on the left hand and in three cases on the right wrist, the prevalence between males and females was four and one, respectively. 

The t-test for statistical significance showed that the variation in Lanz group 4A was significantly more frequent (p<0.01, Table 1).

**Table 1:** Test of the proportion of Lanz group 4A in comparison to all dissected cadavers

Variation		Yes	Total	Portion	p-value
Lanz 4A	Lanz 4A = Yes	19	30	0.633	
	Score	19	30	0.633	<0.01

**Figure 4:**
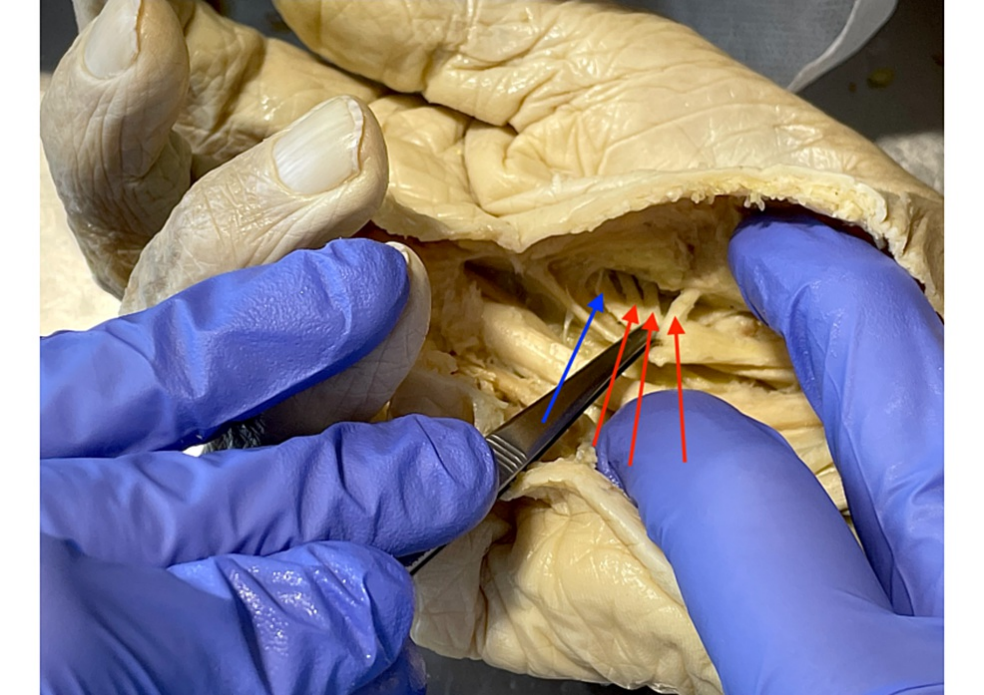
Lanz group 4A with three TMB The blue arrow shows the branch to the index finger, while each of the three red arrows points to one TMB. TMB: thenar motor branch.

Furthermore, the most frequent result (Lanz 4A) was checked for statistical significance between men and women. The calculated p-value was greater than 0.5. There is no statistical significance of the prevalence of Lanz group 4A between men and women. Three arms dedicated to Lanz group 4A showed additional anatomical patterns. In two hands, the lumbrical muscles had their origin in the carpal tunnel. One hand out of 19 (5.2%) had a third TMB (Figure 4). All three previously described anatomical variations of Lanz group 4A were found in the right arms. One female and one male cadaver showed the origin of the lumbrical muscles within the CT. The variation with three TMBs (Figure 4) was found in a male cadaver. The overall distribution of the variation Lanz group 4A between men and women showed rather no differences (Table 2).

**Table 2:** Distribution of dissected cadavers according to sex

Gender	Number of arms	Percent
Female	16	53.3%
Male	14	46.7%
Total	30	100.0%

**Figure 5:**
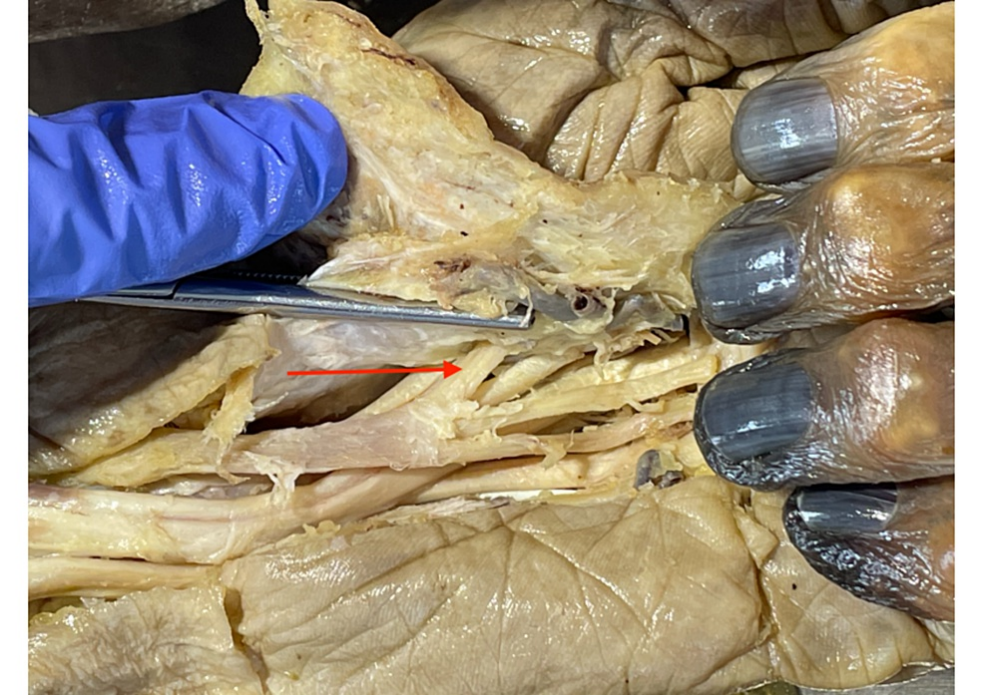
Lanz group 1B Transligamentous TMB (red arrow). TMB: thenar motor branch.

In one male cadaver, a slightly different variation to Lanz group 2 was identified. In the case of Lanz group 2, one of the two TMBs had its origin below the FR instead of distal to it (Figure 5). There were no other anatomical variations found in hands dedicated to Lanz group 2. The variation of Lanz group 2 was neither statistically significantly rare nor significantly frequent.

A transligamentous TMB was found in two hands. The incidence between males and females was equal. In total, the variation dedicated to Lanz group 1B was found in 6.6% of cases (Figure 5). The variations in both hands were described by Lanz and did not show any other anatomical variations. Both variations were found in the left wrist.

**Figure 6:**
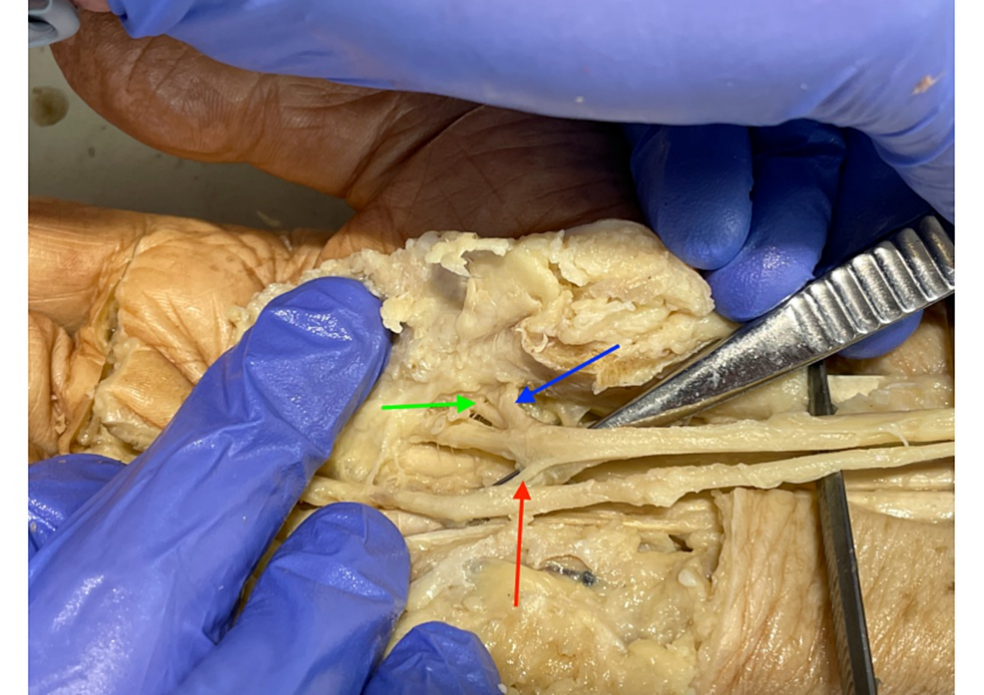
Lanz group 3A Connection branch distal to the FR (red arrow), and two distal TMB (blue and green arrow). TMB: thenar motor branch.

The variation of Lanz group 3A was found in 6.6% of cases (two hands). Figure 6 shows the typical characteristics of Lanz group 3A. A high division of the median nerve with a connection distal to the FR (red arrow) as well as two distal TMB marked by the blue and green arrows. In this case, both variations were found in the right hand in one male and one female cadaver.

**Figure 7:**
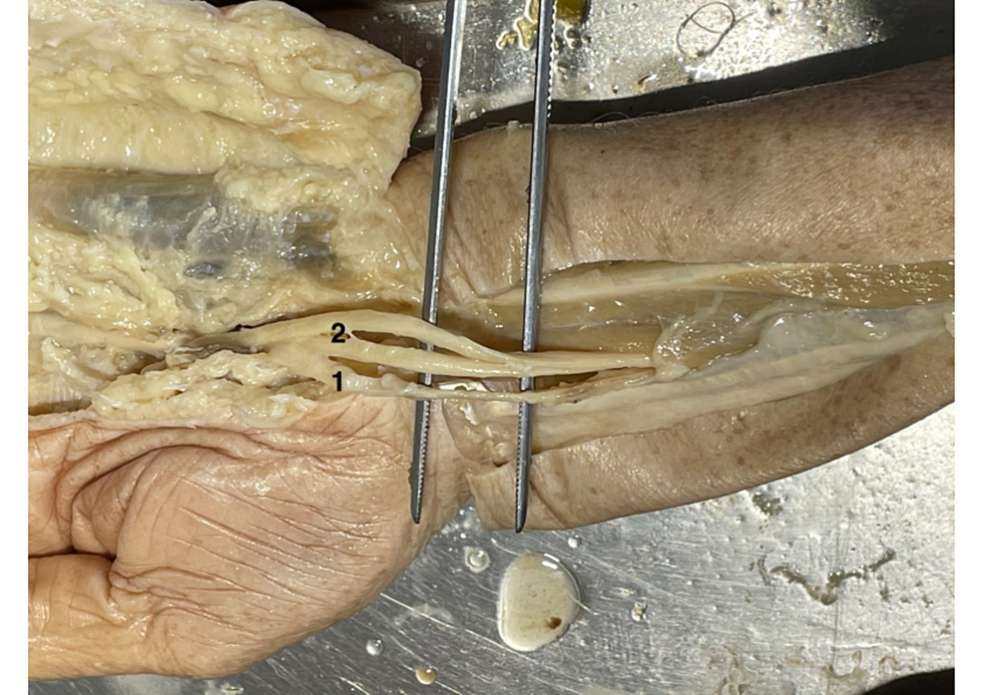
Lanz group 3B with a persistent median artery Lanz group 3B with a persistent median artery; 1) persistent median artery; and 2) bifid median nerve.

**Figure 8:**
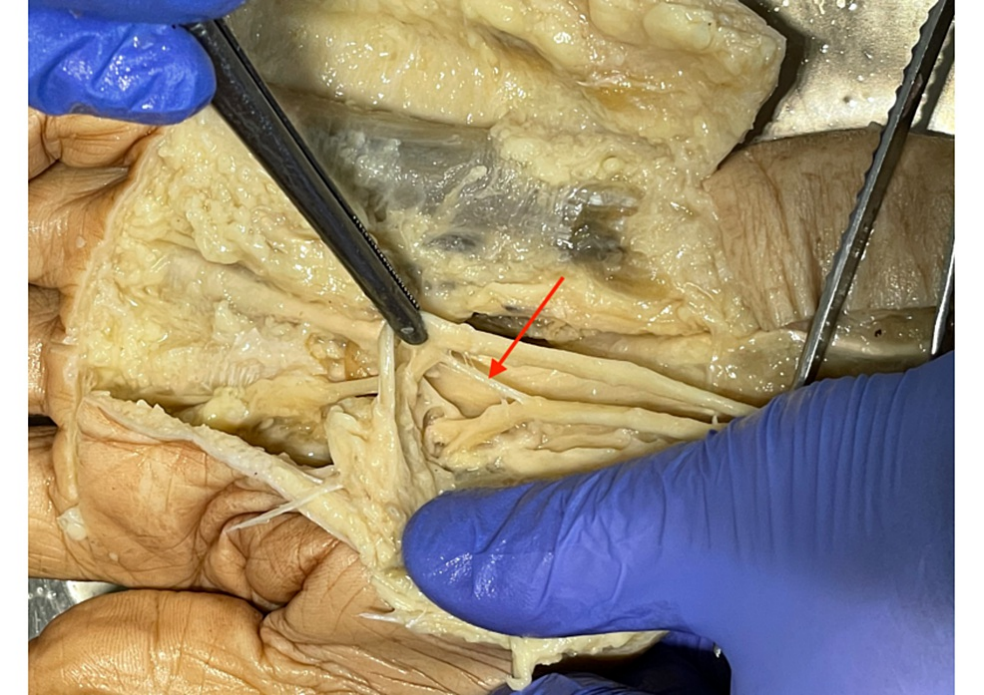
Lanz group 3B with the connection of the bifid median nerve

**Figure 9:**
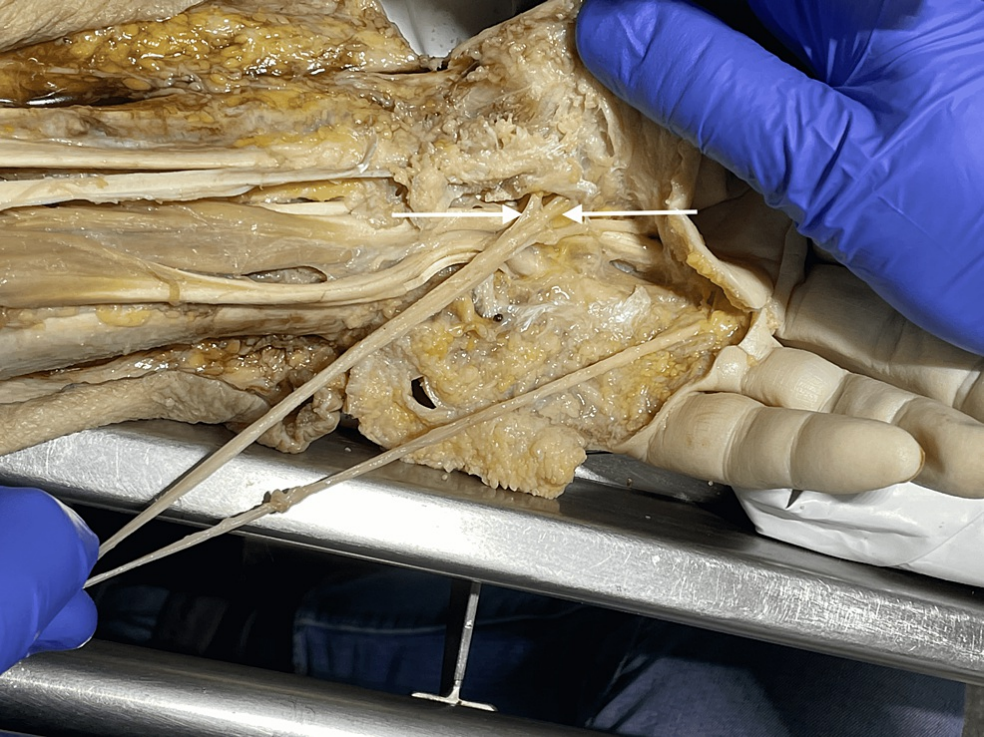
Bifid median nerve with two TMB TMB: thenar motor branch.

One of the least common variations which were found was Lanz group 3B with 3.3% of cases. The characteristic of this variation is a persistent median artery in combination with a high division of the median nerve (Figure 7). The variation was found on the left wrist in a male cadaver. Additionally, the bifid median nerve showed a connection branch distal to the FR (Figures 8, 9). This variation is statistically significantly rare in relation to the rest of the cadavers (p<0.01) (Table 3).

**Table 3:** Test of the proportion of Lanz group 4A

Variation		No	Total	Portion	p-value
Lanz 3B	Lanz 3B = No	29	30	0.967	>0.001
	Score	29	30	0.967	0.000

One hand showed a variation which was not described by Lanz. The variation was found on the right wrist in a female cadaver. The bifid median nerve showed two TMBs. The median nerve had no connection in between. One TMB was passing through the FR, and the other branch was distal to the FR (Figure 9).

In total, the prevalence of transligamentous branching was most common with 73.3%. The second most common branching was the extraligamentous type (26.6%), while a subligamentous branching was not found (Table 4). Bilateral symmetry was found in five cadavers (16.6%). In four cases of bilateral symmetry, the variation was dedicated to Lanz group 4A. In one case, the symmetry found was the variation of Lanz group 2.

**Table 4:** Motor branches to the thenar muscles in relation to the FR FR: flexor retinaculum.

Type	Transligamentous	Extraligamentous	Subligamentous	Total number
Number of hands	22	8	0	30
Percentage	73.3%	26.6%	0%	100.0%

Table 5 compares the most common variation of Lanz group 4A with the second most common Lanz group 2. It shows that also the difference between Lanz group 4A and Lanz group 2 is statistically significant in this study (p<0.01). The difference between all other variations (Lanz group 2 +3) was not statistically significant.

**Table 5:** Comparison of Lanz group 4 and Lanz group 2

	Lanz group 4A	Lanz group 2	Difference
Sample proportion	0.633	0.166	0.467
99% CI asymptotic	0.406 to 0.859	-0.009 to 0.341	0.141 to 0.792
p-value	<0.01		<0.01

## Discussion

The importance of anatomical knowledge of CT is important, and its variations have been described in various studies [6,14,18,19]. Lanz extended the classification of Poisel and added more detailed anatomical features. In this study, 15 cadavers were dissected, and the contents of the CT were compared to the schematic illustrations of Lanz. Furthermore, the results were checked for their statistical significance. On one hand, it was proven that Lanz group 4A was a statistically significant frequent variation. On the other hand, we found that Lanz groups 1B, 3A and 3B and the variation which was not described by Lanz were significantly rare variations. The only variation without any statistical significance in relation to the other results was Lanz group 2.

Since a lot of studies also use Poisel classification for comparison of their results, we also grouped our results due to the classification of Poisel to create a better and more comparable scenario to these studies. In our study, the transligamentous branching in relation to the flexor retinaculum was most common with 73% followed by the extraligamentous type with about 27%. A subligamentous type was not found in any cadaver (Table 4).

Results from other studies are showing a different distribution of variations. Compared to a meta-analysis published by Henry et al. including 31 studies (n=3,918 hands), the pooled prevalence of branches in relation to the FR was 75.2% for extraligamentous, 13.5% for subligamentous and 11.3% for transligamentous course [8]. While the transligamentous branching type was the most frequent variation in our study, it was the least common variation in the meta-analysis including approximately 4,000 hands [8]. Agarwal et al., in their cadaveric study performed in India, found that in 42.3%, a transligamentous type was the most prevalent variation. They included 52 hands in total [14].

Furthermore, the prevalence of Lanz group 4 was least common in the mentioned meta-analysis with 2.3%, while our results showed Lanz group 4A as a statistically most frequent variation with 63.3% [8]. In contrast, the findings for the prevalence of Lanz groups 2 and 3 were rather equal compared to the meta-analysis [8]. Lanz groups 3A and 3B are crucial variations which are associated with carpal tunnel syndrome. If we compare our results to a subgroup analysis of the meta-analysis from Henry et al. based on the geographical region, the prevalence of the extraligamentous, subligamentous and transligamentous types in Europe was 63.7%, 24.6% and 11.7%, respectively [8].

Having a look at all geographical subgroup analysis, the frequency of the subligamentous form is estimated to be 24.6% in Europeans, 12.6% in Asians and 7.1% in Americans. These results are different compared to our study results since we could not find any form of a subligamentous type [8]. In comparison, while the transligamentous type was the most common variation in our study, the pooled prevalence of a transligamentous type was the second most common with 19.4% in the population of the United States of America, but the least common variation in Europe (11.7%) and Asia (8.7%) [8].

Coming back to the classification of Lanz and our most common variation group 4A (63%), only a few studies (n=821 hands) described the variation of Lanz group 4A. In a first subgroup analysis only including cadaveric studies (n=289 hands), the pooled prevalence was 3.1%, and in the subgroup only including intraoperative studies, the prevalence was even lower with 2% (n=532 hands) [8]. These findings differed significantly from our results (Table 6).

**Table 6:** Comparison of Lanz group 4A with other populations

Study	Population (total number of hands)	Lanz group 4A
Lanz (1977) [5]	German (246)	1.63%
Agarwal et al. (2014) [14]	Indian (52)	1.92%
Tountas et al. (1987) [20]	American (821)	2.1%
Alizadeh et al. (2006) [21]	Iranian (60)	18.4%
Steinberg et al. (1998) [16]	Israeli (46)	21.7%
Present study	Lithuanian (30)	63.3%

Steinberg et al. published in a cadaveric study performed in Israel (n=46 hands) that 21.7% showed the variation of Lanz group 4A [16]. These results are higher compared to the meta-analysis but still rather low in comparison to our study (Table 6). Not only the results of Lanz group 4A were different compared to other literature. The general incidence of a high division of the MN described in the literature is between 1% and 3.3% [19]. Asghar et al. published in 2022 that the incidence of a high division of the MN in the Bulgarian population was 0.02% [19]. In 51 dissected cadavers, only one hand showed a bifid MN. Similar findings were published by Ahn et al. in 2000. In a prospective study performed from 1995 till 1997 at the Korea University Anam Hospital, they found that only one hand out of 192 patients (0.3%) showed the anatomical pattern of a high division of the MN [22]. In our study, a high division of the MN was found in 10% of cases and is compared to Bulgaria, Korea, North America, Germany and Poland more frequently. Agarwal et al. described a similar prevalence of 11.5% in the Indian population in 2014 [14]. One of the highest incidences of a high division of the MN was found in Japan [22] (Table 7).

**Table 7:** Comparison of incidence of high division of MN MN: median nerve.

Study	Population (total number of hands)	Observation
Asghar et al. (2022) [19]	Bulgarian (154)	0.02%
Ahn et al. (2000) [22]	Korean (354)	0.3%
Barbe et al. (2005) [23]	North American (89)	2.6%
Lanz (1977) [5]	German (246)	2.8%
Mizia et al. (2011) [18]	Polish (60)	5.0%
Agarwal et al. (2014) [14]	Indian (52)	11.5%
Shinagawa et al. (2019) [24]	Japanese (698)	16.9%
Present study (2022)	Lithuanian (30)	10.0%

Interestingly, the variation of Lanz group 3 is related to a higher incidence of CTS since a bifid MN occupies more space within the CT [25,26]. Less space within the CT may lead to consideration of open-release surgery [26]. Therefore, a bifid MN with a persistent median artery gives an even higher risk for CTS [26].

Another difference while comparing the present study with results from other authors is the frequency of bilateral symmetry. While in our study, only about 16% of all cadavers showed a bilateral symmetry; an analysis including four intraoperative studies of 423 patients undergoing bilateral carpal tunnel release reported a bilateral symmetry in 72.3% of cases. In 27.7%, the course of the TMB was different [8]. The only variation which was not described by Lanz, a bifid median nerve with two thenar branches, was not found in the literature. Only a similar finding was described by Kornberg et al. in 1983. They described a bifid median nerve with three thenar branches in their case report [27].

Limitations of the study

A main limitation of this study may be the evidence of our results. Due to a rather low number of cadavers, the evidence of this study is not very high. Almost all studies we compared our results with included at least or more than 40 patients/cadavers. However, we found some significant differences in the variation in the Lithuanian population. Additionally, a significant difference regarding the prevalence of the type of branching was present in the study itself.

This study may give a good first overview of the distribution of the anatomical variations of Lanz in the Lithuanian population, but further studies should be done to extend our results and thus reach a higher level of evidence. It would be interesting to see whether the distribution of the classifications in a bigger study, either in a cadaveric study or an intraoperative study, would underline the results of the present study.

Suggestions

The knowledge of the anatomy around the wrist is very important before performing surgery to avoid iatrogenic injuries to any structure and especially to the median nerve. Due to its anatomical variations, either number of branches or origin of branching, the median nerve is rather delicate for these injuries. Therefore, the median nerve is still one of the most common nerves affected by iatrogenic injury. The prevalence of anatomical variations varies in different populations. The branching pattern in the Lithuanian population does differ from most populations. This study concludes the most common variations found in the Lithuanian population. Furthermore, the comparison to other populations shows the broad spectrum and individuality of anatomical variations. Therefore, surgeons should keep these possible variations in mind to avoid injury.

In the literature, the ulnar approach to the carpal tunnel is recommended due to a lower chance of injury of the median nerve. Based on the results of this study, we would recommend the ulnar approach as well since we did not find any branching on the ulnar side in 15 cadavers.

## Conclusions

Anatomical variations in the course of the median nerve or its branches are common in the population. In this pilot cadaveric study of anatomical variations of the median nerve in the Lithuanian population, all hands showed different anatomical variations compared to the standard variation of Lanz. The most frequent anatomical variation was in Lanz group 4A (63%). The knowledge of anatomical variations regarding the innervation and course of the median nerve is inevitable, especially in the case of surgery at the wrist (e.g., carpal tunnel surgery).

It is important for hand, orthopedic and neurosurgeons to be aware of these anatomical variations. Since each population seems to have their own incidence of anatomical variations, it is important to know the most frequent ones and possible variations to avoid iatrogenic injury to the nerve. A preoperative diagnostic (e.g., ultrasound) can help to identify patients with a bifid median nerve or a persistent media artery.
